# Successful management of DPP-4 inhibitor-induced bullous pemphigoid with Stapokibart injection in an elderly diabetic patient: a case report

**DOI:** 10.3389/fmed.2026.1755336

**Published:** 2026-03-20

**Authors:** Man Yu, Shue Tian, Fanlin Meng

**Affiliations:** 1 Department of Dermatology, Hospital of Chengdu University of Traditional Chinese Medicine, Chengdu, Sichuan, China; 2 Department of Emergency, Hospital of Chengdu University of Traditional Chinese Medicine, Chengdu, Sichuan, China; 3 Department of Dermatology, Deyang Hospital Affiliated to Hospital of Chengdu University of Traditional Chinese Medicine, Deyang, Sichuan, China

**Keywords:** bullous pemphigoid, diabetes, dipeptidyl peptidase-4 inhibitors, linagliptin, Stapokibart

## Abstract

Dipeptidyl peptidase-4 (DPP-4) inhibitors, such as linagliptin, have been implicated in inducing bullous pemphigoid (BP) in elderly diabetic patients. Stapokibart is a humanized monoclonal antibody targeting interleukin-4 receptor subunit alpha (IL-4Rα), primarily used for atopic dermatitis, with no prior reports of its application in BP. This article describes a case of DPP-4 inhibitor-induced bullous pemphigoid treated with Stapokibart. Following Stapokibart therapy, the patient exhibited complete resolution of skin lesions, significant improvement in quality of life, and no adverse reactions. This case suggests that Stapokibart may be an effective treatment for bullous pemphigoid induced by DPP-4 inhibitors.

## 1 Introduction

The pathogenesis of bullous pemphigoid involves a type 2 immune response, with the cytokines interleukin-4 (IL-4) and interleukin-13 (IL-13) playing a pivotal role. Elevated levels of IL-4 and IL-13 have been demonstrated in the serum and blister fluid of patients with bullous pemphigoid, promoting eosinophil activation, autoantibody production, and inflammatory tissue damage (1).

Stapokibart is a humanized monoclonal antibody targeting the interleukin-4 receptor alpha subunit (IL-4Rα), thereby blocking the signaling pathways of both IL-4 and IL-13. This article reports a novel case of an 87-year-old male with type 2 diabetes who developed widespread blisters and erosions shortly after linagliptin initiation. Diagnosis was confirmed by elevated anti-BP180 antibody titers, histopathological examination, and direct immunofluorescence. The patient was treated with subcutaneous injections of Stapokibart, and complete resolution of skin lesions was achieved within 2 months without recurrence. To our knowledge, this is the first recorded use of Stapokibart for the treatment of DPP-4 inhibitor-induced bullous pemphigoid, suggesting that this drug may represent a novel potential therapeutic option for such diseases.

## 2 Case presentation

**Figure 1:**
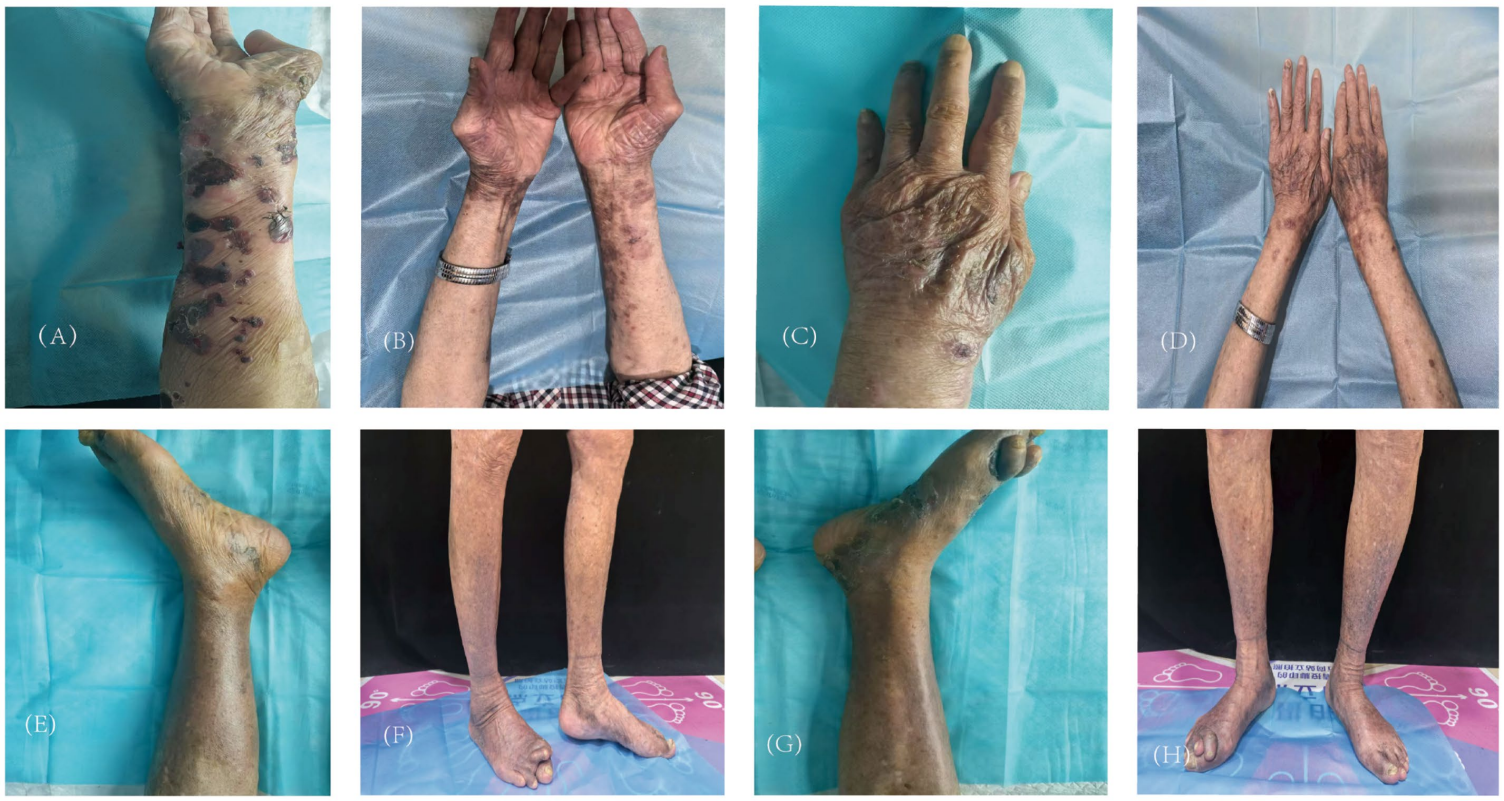
Observe the condition of skin lesions before and after treatment with Stapokibart. **(A,C,E,G)** Before treatment. **(B,D,F,H)** Week 8 of treatment.

**Figure 2:**
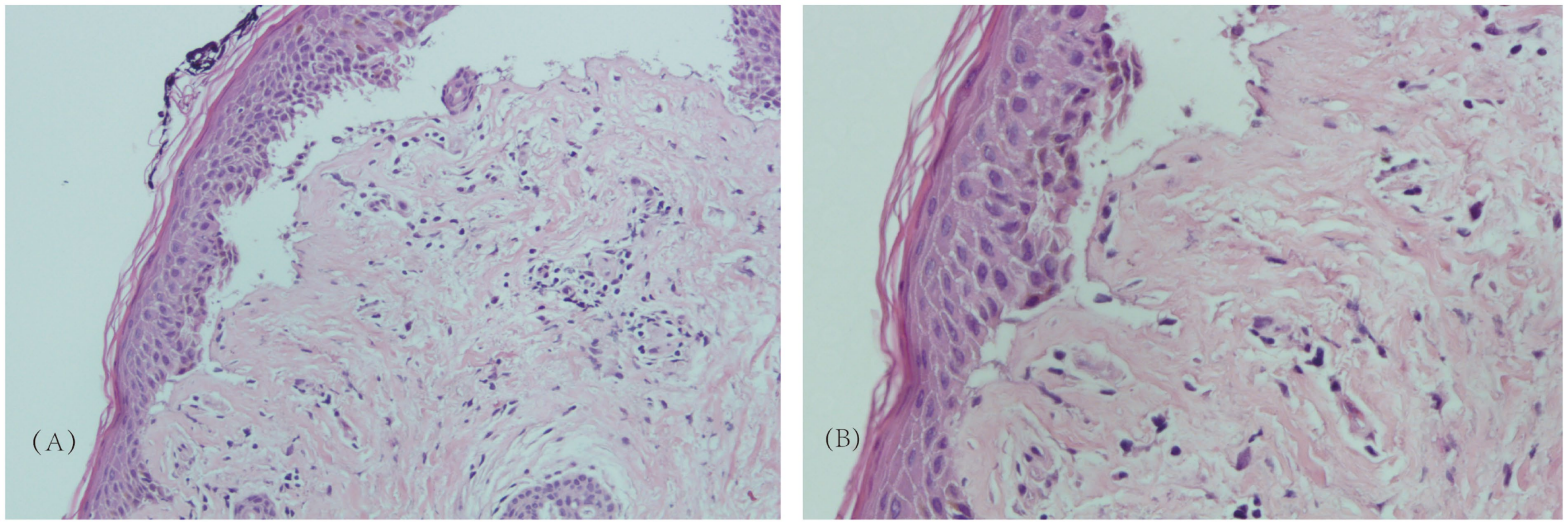
Histopathological results of the skin on the right forearm. **(A)** The epidermis is basically intact, with clefts or bullae formation at the epidermal-dermal junction, presenting as subepidermal bullae. There is a large infiltration of inflammatory cells in the bulla cavity and the dermal papillary layer, primarily eosinophils, accompanied by a small number of lymphocytes and neutrophils, with eosinophil degranulation observed. There is mild to moderate infiltration of lymphocytes and eosinophils around the vessels in the superficial dermis. The dermal papillae show mild edema, and no acantholytic cells are seen (HE × 200). **(B)** The epidermis has intact structure and is separated from the dermis, forming subepidermal bullae. A large number of eosinophils, lymphocytes, and fibrinous exudates are visible in the bulla cavity. There is extensive infiltration of eosinophils and lymphocytes in the dermal papillary layer, accompanied by edema of the dermal papillae. Mild chronic inflammatory cell infiltration is seen around the vessels in the superficial and mid dermis, and no acantholytic cells are observed (HE × 400).

**Figure 3:**
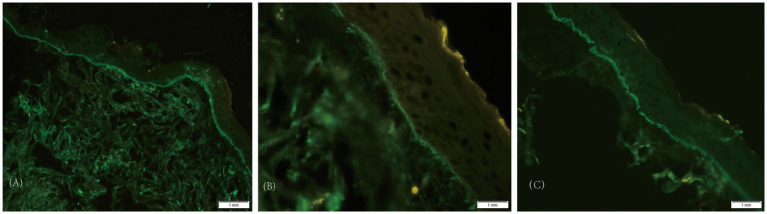
**(A)** Diffuse, network-like immunofluorescent deposits can be seen in the superficial dermis and basement membrane zone. **(B)** The basement membrane zone structure is clearly outlined due to IgG/C3 deposition, appearing as a sharp, bright linear fluorescent band. **(C)** The deposits are continuous with high fluorescence intensity and no obvious interruptions. IgA and IgM tests are negative.

A 87-year-old man presented with erythema and blisters on his limbs that had persisted for half a month. He had a 10-year history of type 2 diabetes mellitus, which was managed with long-term insulin lispro protamine; additionally, linagliptin tablets were added to his treatment regimen 6 months prior. Four years earlier, he was diagnosed with chronic obstructive pulmonary disease (COPD), and he had a 30-year smoking history, averaging approximately 20 cigarettes per day. The onset of his current symptoms began on June 24, 2025, when he developed erythema and blisters on his limbs, accompanied by mild burning pain and severe itching. Some of the blisters had ruptured, exposing red erosions covered with crusts. Over the course of the half-month during which he sought medical care, he was initially diagnosed with “eczema” and treated with olopatadine hydrochloride and calamine lotion; however, there was no improvement in his condition. His medical history was otherwise unremarkable, with no reported infectious diseases, trauma, surgery, drug or food allergies, or family genetic diseases. Upon presentation to our hospital, a physical examination revealed erythema and blisters primarily on both forearms and lower legs, along with some erosions. Both feet were swollen and covered with crusts, and the right foot exhibited deformity of the second and third toes, though this deformity was unrelated to the blisters (Figure 1). The Bullous Pemphigoid Disease Area Index (BPDAI) score at this time was 40. Laboratory test results show: white blood cell count, 9.72 × 10^9/L; eosinophil percentage, 8.1%; eosinophil absolute count, 0.79 × 10^9/L. Total immunoglobulin E (IgE) level is 1430 IU/mL; glycated hemoglobin is 7.1%. Liver function and kidney function tests show no obvious abnormalities. Auxiliary laboratory tests included a pemphigus antibody panel, which showed anti-BP180 antibody levels of 156.11 RU/mL and anti-BP230 antibody levels of 32.35 RU/mL. A skin biopsy performed on the right forearm on July 14, 2025, demonstrated subepidermal blister formation with infiltration of eosinophils and lymphocytes (Figure 2). Immunofluorescence staining results were negative for IgM and IgA but positive for IgG (linear pattern) and C3 (linear pattern), findings consistent with pemphigoid changes (Figure 3). Based on the combination of clinical symptoms, histopathological findings, and immunofluorescence results, the diagnosis was established as dipeptidyl peptidase-4 (DPP-4) inhibitor-associated bullous pemphigoid.

## 3 Therapeutic evaluation

**Figure 4:**
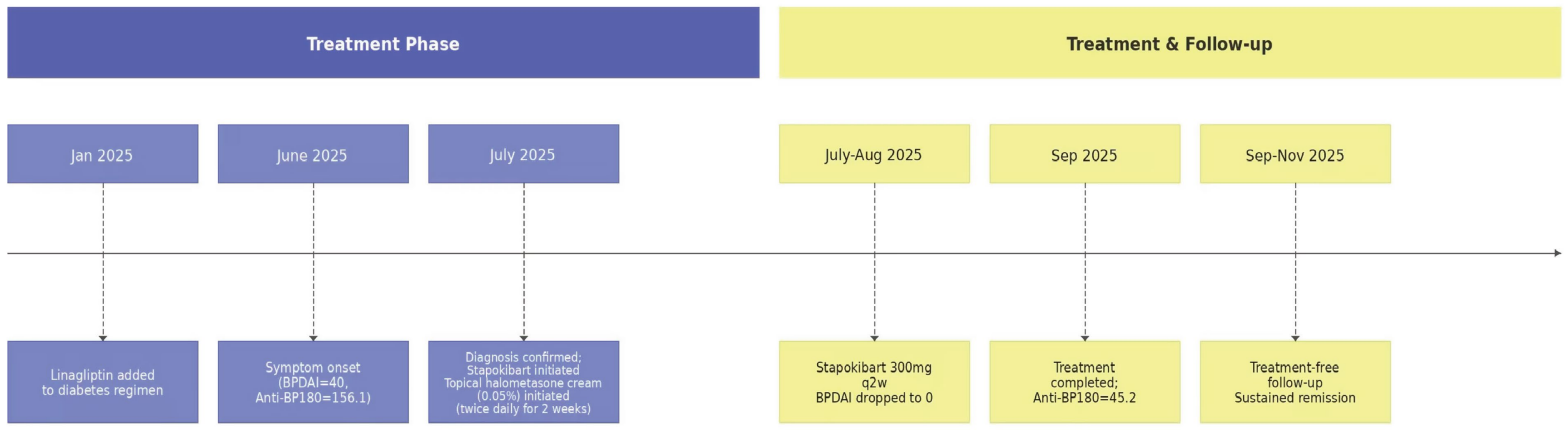
Timeline of clinical course. A graphical timeline summarizing key events: DPP-4 inhibitor initiation, symptom onset, diagnosis, treatment phases with Stapokibart, follow-up visits, and key outcomes including BPDAI scores and antibody trends.

The clinical course is summarized in Figure 4. Following consultation with the endocrinology department, his insulin regimen was adjusted to subcutaneous insulin glargine and insulin aspart, and linagliptin was discontinued. After excluding contraindications and obtaining patient consent, subcutaneous Stapokibart therapy was initiated on July 15, 2025, with an initial dose of 600 mg, followed by 300 mg every 2 weeks. Topical treatment medications, including fusidic acid ointment and halometasone ointment, were also prescribed to utilize their anti-inflammatory effects. The topical treatment was initiated concurrently with the first Stapokibart injection (on July 15, 2025) and continued for 2 weeks. After 2 months of treatment, the skin lesions have completely healed, with no residual itching or new blisters appearing. Only residual pigmentation remains. The patient reported a significant improvement in quality of life, with relief from itching and the ability to sleep through the night. Stapokibart was well-tolerated with no adverse events reported during the treatment period. After the initial two-month course, treatment was discontinued. The patient was followed for an additional 2 months (total follow-up of 4 months from initiation), during which no recurrence of blisters or significant pruritus was observed. The BPDAI score at the final follow-up was 0. Laboratory parameters showed marked improvement (Table 1).

**Table 1:** Key clinical and laboratory parameters before and after Stapokibart treatment.

Parameter	Baseline (pre-treatment)	2 months (after treatment)	4 months (follow-up)
BPDAI score	40	0	0
Anti-BP180 antibody (RU/mL)	156.11	45.20*	38.50*
Anti-BP230 antibody (RU/mL)	32.35	28.10	26.8
Eosinophil count (x10^9/L)	0.79	0.15	0.12
Eosinophil percentage (%)	8.1	2.0	1.8
Total IgE (IU/mL)	1430	350*	300*

* Indicates significant improvement compared to the baseline.

## 4 Discussion

Dipeptidyl peptidase-4 (DPP-4) inhibitors, such as linagliptin, are widely used in the management of type 2 diabetes due to their good safety and efficacy. However, existing evidence suggests that these drugs may be associated with the occurrence of bullous pemphigoid (2, 3). Bullous pemphigoid is an autoimmune blistering disease driven by a type II immune response, commonly occurring in the elderly (4). Clinically, it presents with tense blisters and bullae on the skin, either locally or generalized. Its pathogenesis is closely related to anti-BP180 and anti-BP230 antibodies, which induce the disease through complement activation and non-complement pathways (5). Studies have shown that IL-4 and IL-13 play a key role in the pathogenesis (6). These two cytokines promote the activation and recruitment of eosinophils, which are significantly increased in the lesions and blood of patients with bullous pemphigoid and are associated with tissue damage and blister formation. In addition, IL-4 and IL-13 can stimulate B cells to produce autoantibodies (primarily anti-BP180 and anti-BP230 antibodies), which directly attack the skin basement membrane, leading to blister formation (7). DPP-4 inhibitors have structural similarities to basement membrane proteins (such as BP180 or BP230) (8). This molecular mimicry may lead to the production of cross-reactive autoantibodies, thereby triggering an autoimmune response. Furthermore, these drugs may modulate the immune microenvironment to promote exposure of basement membrane antigens, enhance antigen presentation, and increase inflammatory cell infiltration, ultimately resulting in blister formation. Elevated levels of anti-BP180 and anti-BP230 antibodies further support the pathogenic process mediated by autoantibodies (9).

Although discontinuation of the inciting medication is the standard management for drug-induced bullous pemphigoid, glucocorticoids are still considered first-line treatment drugs for bullous pemphigoid. However, after clinical improvement, a gradual reduction in dosage is required, during which patients not only face the risk of recurrence but may also develop adverse reactions due to long-term use of glucocorticoids. In this case, the patient has comorbid diabetes, and glucocorticoids may interfere with glycemic control. Therefore, after excluding contraindications and obtaining patient and family consent, biologic agents were considered for treatment. Biologic agents such as rituximab, omalizumab, and dupilumab have shown potential efficacy in refractory BP patients and can reduce the side effects associated with traditional immunosuppressive therapy (10, 11).

Stapokibart is a humanized IgG1 monoclonal antibody that targets the interleukin-4 receptor alpha (IL-4Rα) (12). By blocking IL-4Rα, a shared receptor component for IL-4 and IL-13, Stapokibart simultaneously inhibits the signaling of both cytokines. Theoretically, this drug may reduce the production of pathogenic autoantibodies and suppress eosinophil-mediated inflammation by inhibiting the IL-4/IL-13 pathway, thereby controlling the course of bullous pemphigoid. Stapokibart has been approved in China for the treatment of adult atopic dermatitis, chronic sinusitis with nasal polyps, and seasonal allergic rhinitis (13). It is important to note that Stapokibart has not been approved for the treatment of bullous pemphigoid by the U.S. Food and Drug Administration (FDA) or other major regulatory agencies outside of China.

Although Stapokibart and Dupilumab are both monoclonal antibodies targeting IL-4Rα, they differ in species cross-reactivity, binding epitopes, and preclinical properties. Studies show that Stapokibart can bind to IL-4Rα in humans, cynomolgus monkeys, and rats, whereas Dupilumab only specifically binds to human IL-4Rα; their epitope binding regions are different. *In vitro* experiments indicate that Stapokibart is comparable to or more potent than Dupilumab in blocking the IL-4Rα signaling pathway (14). Previous literature reports that Dupilumab can improve the condition of patients with bullous pemphigoid (15, 16). Given that this case patient exhibited significantly elevated IgE and eosinophil levels, and bullous pemphigoid is associated with type 2 inflammatory mechanisms, it is hypothesized that Stapokibart may treat this disease through a similar mechanism—namely, by blocking the type 2 inflammatory pathway.

Compared to traditional treatments such as systemic glucocorticoids or immunosuppressants, Stapokibart has significant advantages. It works faster—in this case showing efficacy within 2 months—and can avoid the side effects associated with long-term use of glucocorticoids, such as worsened hyperglycemia, osteoporosis, or increased risk of infection. These benefits make it particularly suitable for elderly diabetic patients.

### 4.1 Strengths and limitations

The main strength of this report is the novel documentation of Stapokibart’s efficacy in a clinically challenging scenario of DPP-4 inhibitor-induced BP, with detailed objective and subjective outcome measures. However, this study has several limitations. As a single-case report, it cannot establish efficacy or generalizability. The follow-up period of 4 months, while extended from the initial 2 months, is still relatively short for assessing long-term relapse risk and rare adverse events. Furthermore, the absence of post-treatment biopsy or serial antibody titers beyond the reported timepoints limits the mechanistic insights into the sustained response. The findings are exploratory and should be interpreted as hypothesis-generating for future larger-scale studies.

## 5 Conclusion

This case report describes the successful treatment of bullous pemphigoid with Stapokibart. It emphasizes the crucial importance of promptly identifying and discontinuing the causative drug in the management of drug-induced bullous pemphigoid (BP). Additionally, Stapokibart offers a precise and safe alternative, reducing reliance on traditional glucocorticoids. This approach is particularly beneficial for elderly patients with multiple comorbidities, as it optimizes treatment strategies and improves outcomes. Based on a review of relevant domestic and international literature, this is the first reported case of bullous pemphigoid treated with Stapokibart. However, this is a single-case report with a short follow-up period, which may not be sufficient to fully evaluate long-term efficacy, recurrence risk, or rare adverse reactions. Further clinical research is needed to validate the efficacy and safety of Stapokibart in treating bullous pemphigoid.

## Data Availability

The raw data supporting the conclusions of this article will be made available by the authors, without undue reservation.
